# Discriminating Osteoarticular Infection Bacterial Aetiology Using Immune–Inflammatory Biomarkers

**DOI:** 10.3390/microorganisms14091959

**Published:** 2026-09-04

**Authors:** Matteo Fortunato, Anne Tabard-Fougère, Elio Paris, Giacomo De Marco, Oscar Vazquez, Christina Steiger, Ardian Ramadani, Andreas Tsoupras, Romain Dayer, Dimitri Ceroni

**Affiliations:** 1 Faculty of Medicine, University of Geneva, 1206 Geneva, Switzerland; 2 Paediatric Orthopaedics Service, Geneva Children’s Hospital, Geneva University Hospitals, 1211 Geneva, Switzerland

**Keywords:** *Kingella kingae*, paediatric osteoarticular infection, pyogenic infection, immune–inflammatory biomarkers, diagnostic biomarkers

## Abstract

Identifying the causative pathogen of paediatric osteoarticular infections (OAIs) is essential for guiding management. *Kingella kingae* infections are generally milder than pyogenic infections, which may have more severe and lasting consequences. Because standard inflammatory markers have limited discriminatory value, we assessed whether complete blood count-derived immune–inflammatory biomarkers (IIBs) could provide a simple, low-cost diagnostic aid. We retrospectively reviewed children younger than 5 years admitted to a tertiary hospital with confirmed OAIs. *K. kingae* cases were included from 2007 to 2025 and pyogenic cases from 1997 to 2025. Admission clinical data, conventional inflammatory markers, and six IIBs—NLR, MLR, PLR, SII, SIRI, and PIV—were analysed. The cohort included 118 *K. kingae* and 39 pyogenic OAIs. Pyogenic infections were associated with higher admission temperature and CRP levels and lower lymphocyte counts. All six IIBs were significantly increased in pyogenic infections and showed excellent univariable discrimination, with AUCs of 0.92–0.94. In a common complete-case cohort, the clinical model incorporating age, admission temperature, and CRP achieved an optimism-corrected AUC of 0.848. Adding lymphocyte count or an individual IIB increased corrected AUCs to 0.951–0.962; apparent AUCs were significantly higher than that of the clinical model after Holm adjustment. The abnormal-IIB-count model achieved the highest corrected AUC of 0.970; apparent sensitivity and specificity at the Youden-optimal threshold were 97.3% and 90.3%, respectively, but it did not significantly outperform the model with lymphocyte count alone. Routine CBC-derived IIBs may improve early differentiation between *K. kingae* and pyogenic OAIs, although external validation is required before clinical implementation.

## 1. Introduction

Paediatric osteoarticular infections (OAIs) are potentially serious conditions that can lead to septic complications, impaired bone growth, joint destruction, and long-term functional impairment [1]. Their presentation is variable and may involve multiple musculoskeletal structures. In addition to acute haematogenous osteomyelitis, septic arthritis, and spondylodiscitis, paediatric OAIs can also have fewer common presentations, including pyomyositis, tendon sheath infections, and bursitis.

Since the early 2000s, nucleic acid amplification assays have markedly improved microbiological diagnosis and refined our understanding of the aetiology and pathophysiology of paediatric OAIs, prompting major changes in diagnosis and treatment [2]. Pyogenic infections, particularly those caused by *Staphylococcus aureus* (*S. aureus*) or *streptococci*, are generally more severe, resolve more slowly, and lead to poorer outcomes, supporting invasive diagnostic procedures and early intravenous (IV) antibiotic therapy [3,4]. Conversely, OAIs caused by *Kingella kingae* (*K. kingae*) usually have a milder course and can often be managed successfully with oral antibiotics alone [5]. *K. kingae* peaks between 6–48 months and causes 50–80% of culture-positive OAIs in children under 5 years, where molecular detection is routine, whereas pyogenic pathogens predominate in older children [5]. Therefore, early differentiation between pyogenic OAIs and *K. kingae* infections is essential at this young age group. Moreover, OAIs caused by *K. kingae* and pyogenic pathogens have distinct clinical and biological profiles. Although conventional inflammatory markers may support diagnosis, they are generally insufficient to reliably identify the causative microorganism.

Recently, complete blood count (CBC)-derived immune–inflammatory biomarkers (IIBs) have gained attention as simple, low-cost, and readily available indicators of systemic inflammation. They have been evaluated in several medical specialties for diagnosis, monitoring disease activity, and assessing prognosis [6,7,8,9,10,11,12,13]. However, their reference ranges vary with age, sex, and population characteristics, and most evidence comes from adult cohorts rather than healthy young children [14,15,16,17,18,19,20]. Paediatric data, therefore, remain limited, and their usefulness in childhood OAIs has not been clearly defined. To date, no study has systematically evaluated CBC-derived immune–inflammatory biomarkers to distinguish *K. kingae* from pyogenic OAIs in young children.

Thus, this study aimed to determine whether CBC-derived immune–inflammatory biomarkers enhance discrimination between OAIs caused by *K. kingae* and those caused by pyogenic bacterial pathogens, particularly MSSA and streptococci, in children younger than 5 years. In an exploratory secondary analysis, we also assessed whether lymphocyte count and IIBs provided additional discriminatory information beyond age, admission temperature, and CRP.

## 2. Materials and Methods

### 2.1. Study Design and Setting

After approval by the Children’s Hospital Ethics Review Committee (CER 2023-00578), we retrospectively reviewed the medical records of all children younger than 5 years admitted to our hospital with OAIs. This age restriction ensures optimal comparison between *K. kingae* and pyogenic OAIs, while minimising age-related confounding in CBC-derived biomarker interpretation.

### 2.2. Population and Case Definitions

Children with confirmed *K. kingae* OAI cases were included from January 2007, when routine molecular detection of *K. kingae* was implemented, through 2025, while pyogenic bacterial OAI cases were reviewed from 1997 to 2025. The criteria established by Morrey and Morrissey were used to estimate children’s risk of having a bone or joint infection [21,22,23,24]. OAI diagnosis and bacterial aetiology were confirmed when culture or PCR testing of blood samples and/or biopsy samples from infected sites were positive, together with positive magnetic resonance imaging (MRI) [25] findings or standard radiographs.

### 2.3. Data Collection

For each patient, age at diagnosis, sex, temperature at admission, and anatomical site of infection were collected. Patients were also categorised by infection type as septic arthritis (SA), acute or subacute haematogenous osteomyelitis, arthritis with associated osteomyelitis, spondylodiscitis, or other OAI presentations, including pyomyositis and tenosynovitis. Laboratory investigations included C-reactive protein (CRP) value, erythrocyte sedimentation rate (ESR), and complete differential white blood cell count. Patients with missing values for white blood cell counts, neutrophil count, lymphocyte count, monocyte count, or platelet count were excluded. Immune–inflammatory biomarkers were then calculated from absolute blood cell counts at admission. NLR (neutrophil-to-lymphocyte ratio) was defined as neutrophils/lymphocytes, MLR (monocyte-to-lymphocyte ratio) as monocytes/lymphocytes, PLR (platelet-to-lymphocyte ratio) as platelets/lymphocytes, SII (systemic immune–inflammation index) as platelets × neutrophils/lymphocytes, SIRI (systemic inflammation response index) as neutrophils × monocytes/lymphocytes, and PIV (pan-immune–inflammation value) as neutrophils × monocytes × platelets/lymphocytes. All calculations were performed consistently using cell counts expressed as G/L (10^9^ cells/L). NLR, MLR, and PLR were treated as dimensionless ratios. SII, SIRI, and PIV were treated as scale-dependent composite indices rather than dimensionless ratios. Published paediatric reference data were used to contextualise age-related CBC-derived values [20,26,27,28].

### 2.4. Microbiological Investigations

The causative microorganism was systematically investigated using blood cultures. Before 2009, BACTEC 9000 blood culture media(Becton Dickinson Diagnostic Instrument Systems, Sparks, MD, USA) were used; thereafter, samples were analysed with the automated BD BACTEC FX system. Joint fluid and bone aspirate sampling was performed in the operating room under general anaesthesia, usually immediately after MRI. Joint fluid was obtained by aspiration under fluoroscopic guidance. Bone samples were obtained by fluoroscopically guided drilling into the affected bone followed by aspiration of the bone contents. Bone aspirates or joint fluid samples used for Gram staining were prepared and heat-fixed on a glass slide, stained with crystal violet for 1 min, treated with Gram’s iodine for 1 min, decolorized with 95% ethanol for 5 to 10 s, and counterstained with safranin for 1 min. Cell counting was performed manually using a hemocytometer grid. Separate specimens were inoculated onto Columbia blood agar and CDC anaerobe 5% sheep blood agar under anaerobic conditions, onto chocolate agar in a CO_2_-enriched atmosphere, and into brain–heart infusion broth. All media were incubated for 10 days at 35 °C ± 2 °C.

When standard cultures were negative, molecular testing was also performed. Broad-range 16S rRNA gene PCR was performed using BAK11w, BAK2, and BAK533r primers (Eurogentec, Seraing, Belgium). From 2007 onward, real-time PCR targeting the *K. kingae* RTX toxin locus was also applied to peripheral blood and/or biopsy specimens from infected sites [2]. Since September 2009, oropharyngeal swab PCR has also been performed, as the detection of *K. kingae* RTX toxin genes in the oropharynx was considered strongly supportive of *K. kingae* OAI, whereas their absence made this diagnosis unlikely [29].

### 2.5. Statistical Analysis

All statistical analyses were performed using R software (version 4.4.1; R Foundation for Statistical Computing, Vienna, Austria) and the RStudio interface (version 2024.09.0; Posit Software, PBC, Boston, MA, USA). The level of significance was set at *p* < 0.05. As this retrospective study included all eligible cases available during the study period, no a priori sample-size calculation was used to determine cohort size. A retrospective ROC-based power analysis was performed for the six IIBs using the observed sample sizes, testing the observed AUCs against the null hypothesis AUC = 0.50 at α = 0.05 and estimating the AUC detectable with 80% and 90% power.

Categorical variables were presented as frequencies and percentages, and continuous variables as medians with interquartile ranges (IQR) or means with standard deviations (SD), as appropriate. Normality was assessed using the Shapiro–Wilk test. Comparisons between *K. kingae* and pyogenic OAI groups were performed using the Mann–Whitney U test for continuous variables and Fisher’s exact test or chi-square test for categorical variables, as appropriate. Effect sizes for Wilcoxon rank-sum comparisons were expressed as rank-biserial correlations calculated directly from the Mann–Whitney U statistic; positive values indicate higher values in the pyogenic group and negative values indicate lower values.

Receiver operating characteristic (ROC) curve analysis was performed to evaluate the discriminatory performance of classical inflammatory markers and immune–inflammatory biomarkers. The area under the curve (AUC) with 95% confidence intervals (CI) was calculated for each parameter. Optimal cut-off values were determined using the Youden index (sensitivity + specificity − 1). Sensitivity and specificity were calculated for each optimal cut-off. For the primary analysis, CRP values reported only as “<10 mg/L” or “<3 mg/L” in the electronic patient records were assigned half the corresponding reporting limit (5 mg/L and 1.5 mg/L, respectively). A sensitivity analysis assigned the full lower reporting limit (10 mg/L and 3 mg/L, respectively).

To assess whether CBC-derived parameters provided discriminatory information beyond conventional clinical variables, multivariable logistic regression models were fitted with pyogenic infection coded as the positive outcome. The clinical model was defined as age in months, admission temperature, and CRP. Extended models separately added lymphocyte count or one of the six IIBs; the six IIBs were not entered simultaneously. Because the IIB distributions were strongly right-skewed, they were entered as continuous variables after natural-log transformation using ln(1 + value). An exploratory model included the number of IIBs exceeding their individual Youden-derived thresholds. The abnormal-IIB count was calculated using the six thresholds and ranged from 0 to 6. All models were evaluated in the same complete-case population. Model discrimination was assessed using the AUC with 95% confidence intervals, and sensitivity and specificity were calculated at the Youden-optimal predicted-probability threshold. Optimism-corrected AUCs were estimated using 1000 bootstrap repetitions. Because the six IIB thresholds were derived from the study cohort, internal validation of the abnormal-IIB-count model repeated threshold selection and count construction within each bootstrap resample. Apparent AUCs were compared using paired DeLong tests, with the Holm method used to account for multiple comparisons within each comparison family. To assess potential temporal bias, the univariable ROC analyses and multivariable models were repeated after restricting both groups to the common 2007–2025 recruitment period.

## 3. Results

### 3.1. Study Population

During the electronic medical-record search, 322 potential cases of interest were identified, comprising 164 pyogenic OAIs and 158 confirmed *K. kingae* OAIs. Among the 164 pyogenic cases, 30 were excluded because of incomplete WBC data and 95 of the remaining 134 cases because the patients were aged ≥5 years, leaving 39 patients in the final analysis. Among the 158 confirmed *K. kingae* cases, 39 were excluded because of incomplete WBC data and one of the remaining 119 cases because the patient was aged ≥5 years, leaving 118 patients in the final analysis. Complete WBC, neutrophil, lymphocyte, monocyte, and platelet data were available for all 157 patients. Admission temperature was available in 113/118 *K. kingae* and 37/39 pyogenic cases, CRP in 118/118 and 39/39 cases, and ESR in 97/118 and 26/39 cases, respectively.

### 3.2. Epidemiology of OAIs

Table 1 summarises patient characteristics. Children with *K. kingae* OAIs were younger than those with pyogenic infections (17.7 ± 8.4 vs. 24.7 ± 18.5 months). *K. kingae* infections peaked between 12 and 18 months (29.7%), with no cases in infants younger than 3 months. In contrast, pyogenic infections showed no age-related peak. The recorded pyogenic aetiologies comprised *S. aureus*/MSSA (*n* = 19), *S. pyogenes*/group A streptococci (*n* = 12), *S. pneumoniae* (*n* = 6), and *S. mitis* (*n* = 2).

Because OAI categories were not mutually exclusive, 121 OAI manifestations were recorded among the 118 *K. kingae* patients and 47 among the 39 pyogenic patients. Septic arthritis was the leading presentation in *K. kingae* infections (57%, 69/121 cases), most commonly affecting the knee (64%, 44/69 of SA cases), followed by osteomyelitis (19%, 23/121 cases). Pyogenic infections more commonly presented as osteomyelitis (32%, 15/47), with 73% (11/15) involving long bones, followed by septic arthritis (28%, 13/47).

As expected, microbiological yield was low for conventional but high for molecular methods. For *K. kingae* infections, blood and biopsy cultures were positive in 9.5% (9 of 95 patients) and 8.9% (10 of 112 patients) of cases, respectively. Oropharyngeal swab testing for *K. kingae* RTX toxin genes showed a high positivity rate, with 92 of 96 (95.8%) testing positive. PCR performed on biopsy specimens or blood samples was positive in 113 of 113 cases (100%). In the pyogenic group, cultures were positive in 9 of 37 blood samples (24.3%) and in 29 of 37 biopsies (78.4%). Broad-range PCR assays were positive in 14 of 22 cases (63.6%).

### 3.3. Classical Inflammatory Markers

**Figure A1:**
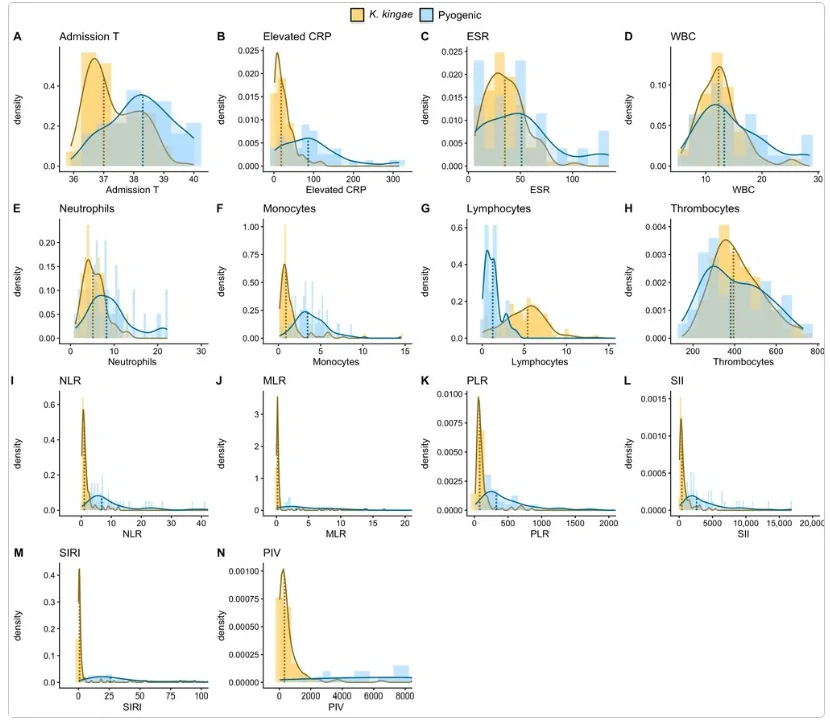
Distributions and medians (dashed lines) for all markers in *K. kingae* and pyogenic OAIs. Subfigures: (**A**) admission temperature; (**B**) C-reactive protein (CRP); (**C**) erythrocyte sedimentation rate (ESR); (**D**) white blood cell count (WBC); (**E**) neutrophils; (**F**) monocytes; (**G**) lymphocytes; (**H**) thrombocytes; (**I**) neutrophil-to-lymphocyte ratio (NLR); (**J**) monocyte-to-lymphocyte ratio (MLR); (**K**) platelet-to-lymphocyte ratio (PLR); (**L**) systemic immune-inflammation index (SII); (**M**) systemic inflammation response index (SIRI); and (**N**) pan-immune-inflammation value (PIV). Admission temperature is expressed in °C; CRP in mg/L; ESR in mm/h. All cell counts are expressed in G/L (10⁹ cells/L).

Children with *K. kingae* OAIs presented with significantly milder inflammatory responses (Table 2; Figure A1). Median CRP was approximately fivefold lower in *K. kingae* versus pyogenic infections (18.3 vs. 86.0 mg/L), and median temperature was significantly lower in *K. kingae* infections (37.0 °C vs. 38.3 °C). Total WBC count was higher in pyogenic OAIs than in *K. kingae* infections (median 13.3 vs. 12.3 G/L), but this difference was not significant and the effect size was small. Neutrophil, monocyte, and lymphocyte counts differed significantly between the two groups. Lymphocyte count emerged as the strongest discriminator (AUC = 0.93, Table 3) with an optimal cut-off of 2.98 G/L (sensitivity 95%, specificity 83%). Lymphocyte count was 4-fold lower in pyogenic infections (median 1.25 vs. 5.38 G/L, *p* < 0.001). Monocyte count (AUC 88%) and CRP (AUC 83%) also showed good discrimination. ESR and platelet counts did not differ significantly between groups.

### 3.4. Immune–Inflammatory Biomarkers

All six CBC-derived biomarkers (NLR, MLR, PLR, SII, SIRI, PIV) were significantly elevated in the pyogenic group compared with *K. kingae* infections (*p* < 0.001, Table 2). ROC curve analysis demonstrated excellent discriminatory performance for all six biomarkers, with AUCs ranging from 92 to 94% (Table 3).

**Figure 1:**
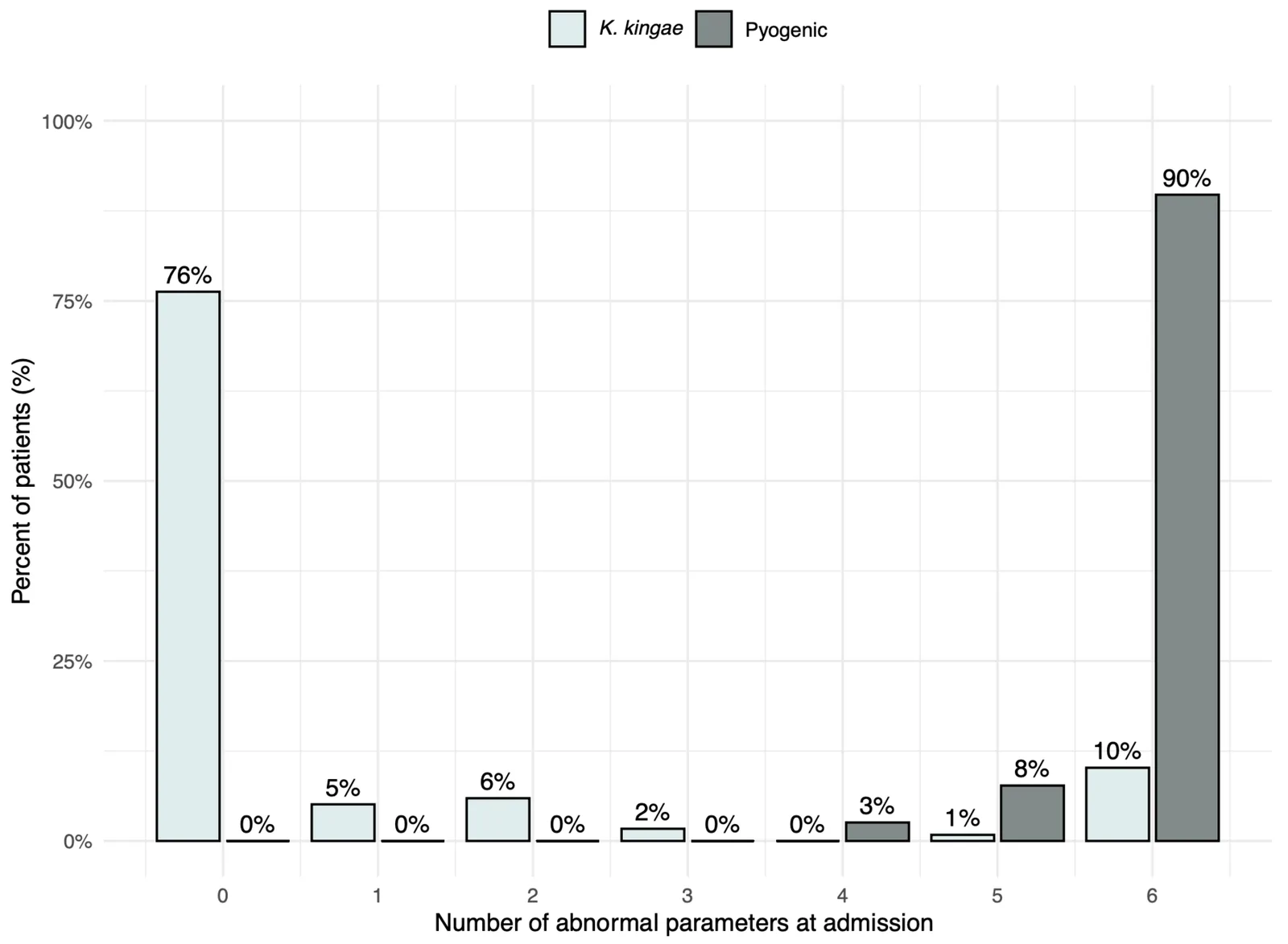
Number of abnormal IIBs present at admission in percentage for *K. kingae* and pyogenic OAIs with established cut-offs.

Figure 1 illustrates the clinical utility of these biomarkers. In the pyogenic group, 90% of patients had all six biomarkers elevated, and none had one or fewer abnormal biomarkers. Conversely, 76% of *K. kingae* OAIs had no abnormal biomarkers, and only 10% had all six biomarkers elevated. In this cohort, the absence of elevated IIBs was more commonly associated with *K. kingae* infection, whereas multiple elevated biomarkers were more commonly associated with pyogenic infection. When considered as an ordinal diagnostic marker, the number of abnormal IIBs showed excellent discrimination, with an AUC of 0.943 (95% CI 0.913–0.974). A threshold of at least four abnormal IIBs maximised the Youden index, yielding 100% sensitivity and 89.0% specificity for pyogenic infection.

**Table 3:** AUCs and cut-off values for classical inflammatory markers and IIBs for children below the age of 5.

Marker	AUC (95% CI)	Cut-Off	*K. kingae*	Pyogenic	Sp/Se
Admission T	76% [67.2 to 85.2%]	≤37.55	71	8	Sp = 63%; Se = 78%
		>37.55	42	29	
CRP	83% [73.4 to 91.6%]	≤53.5	107	12	Sp = 91%; Se = 69%
		>53.5	11	27	
ESR	61% [45.8 to 75.5%]	≤50.5	76	12	Sp = 78%; Se = 54%
		>50.5	21	14	
WBC	59% [47.9 to 70.6%]	≤17.25	107	26	Sp = 91%; Se = 33%
		>17.25	11	13	
Neutrophils	74% [64.3 to 84.0%]	≤7.81	101	16	Sp = 86%; Se = 59%
		>7.81	17	23	
Monocytes	88% [81.7 to 93.9%]	≤1.93	100	3	Sp = 85%; Se = 92%
		>1.93	18	36	
Lymphocytes	93% [88.5 to 96.6%]	≤2.98	20	37	Sp = 83%; Se = 95%
		>2.98	98	2	
Thrombocytes	54% [42.2 to 65.5%]	≤319.5	24	16	Sp = 80%; Se = 41%
		>319.5	94	23	
NLR	94% [90.5 to 97.5%]	≤2.82	102	1	Sp = 86%; Se = 97%
		>2.82	16	38	
MLR	92% [87.9 to 96.6%]	≤0.49	103	0	Sp = 87%; Se = 100%
		>0.49	15	39	
PLR	92% [87.8 to 96.1%]	≤147.29	98	2	Sp = 83%; Se = 95%
		>147.29	20	37	
SII	93% [89.3 to 96.7%]	≤913.15	93	1	Sp = 79%; Se = 97%
		>913.15	25	38	
SIRI	93% [88.9 to 97.1%]	≤5.63	105	0	Sp = 89%; Se = 100%
		>5.63	13	39	
PIV	93% [88.8 to 96.9%]	≤2595.76	104	1	Sp = 88%; Se = 97%
		>2595.76	14	38	

Note: Admission T, admission temperature (°C); CRP, C-reactive protein (mg/L); ESR, erythrocyte sedimentation rate mm/h; WBC, white blood cell count; NLR, neutrophil-to-lymphocyte ratio; MLR, monocyte-to-lymphocyte ratio; PLR, platelet-to-lymphocyte ratio; SII, systemic immune–inflammation index; SIRI, systemic inflammation response index; PIV, pan-immune–inflammation value; Se, sensitivity; Sp, specificity.

### 3.5. Incremental Value of CBC-Derived Parameters

Multivariable models were evaluated in a common complete-case population of 150 children, including 37 pyogenic and 113 *K. kingae* infections. Seven patients (2 pyogenic and 5 *K. kingae*) were excluded from these models because admission temperature was unavailable. The clinical reference model incorporating age, admission temperature, and CRP showed good discrimination, with an apparent AUC of 0.863 (95% CI 0.781–0.946) and an optimism-corrected AUC of 0.848.

Adding lymphocyte count or an individual IIB increased apparent AUCs to 0.959–0.968 and optimism-corrected AUCs to 0.951–0.962 (Table 4). All eight extended models had significantly higher apparent AUCs than the clinical model (age, admission temperature, and CRP) after Holm adjustment (adjusted *p* = 0.016–0.019; Table A1). The model incorporating lymphocyte count achieved an apparent AUC of 0.968 (95% CI 0.945–0.991) and an optimism-corrected AUC of 0.961, with apparent sensitivity of 94.6% and apparent specificity of 89.4% at the Youden-optimal threshold.

The model incorporating the number of abnormal IIBs showed the highest observed performance, with an apparent AUC of 0.977 (95% CI 0.959–0.995), an optimism-corrected AUC of 0.970; apparent sensitivity and specificity at the Youden-optimal threshold were 97.3% and 90.3%, respectively. It correctly identified 36 of 37 pyogenic infections and 102 of 113 *K. kingae* infections. Each additional abnormal IIB was associated with approximately threefold higher adjusted odds of pyogenic infection (OR 3.10, 95% CI 1.71–5.61). Although the abnormal-IIB-count model had the highest observed AUC, it did not significantly outperform the simpler lymphocyte-count model after Holm adjustment (adjusted *p* = 0.831). Results were materially unchanged when values below the reporting limit were assigned either half the reporting limit or the full reporting limit. In the temporal sensitivity analysis restricted to the common 2007–2025 period, 145 children were retained (118 *K. kingae* and 27 pyogenic infections). The six IIBs remained stable, with AUCs of 0.921–0.944 compared with 0.919–0.940 in the primary analysis. In the common complete-case temporal cohort of 138 children (113 *K. kingae* and 25 pyogenic infections), optimism-corrected AUCs were 0.779 for the clinical model, 0.931–0.950 for models adding lymphocyte count or an individual IIB, and 0.954 for the abnormal-IIB-count model. All eight extended models remained significantly superior to the clinical model after Holm adjustment (adjusted *p* = 0.015), while the abnormal-IIB-count model did not significantly outperform the lymphocyte-count model (adjusted *p* = 1.000). For the six IIBs, retrospective ROC power against the null hypothesis AUC = 0.50 was approximately 100%; with 39 pyogenic and 118 *K. kingae* cases, the detectable AUC was 0.645 at 80% power and 0.668 at 90% power (α = 0.05).

## 4. Discussion

In this retrospective cohort of children younger than 5 years with microbiologically confirmed OAI, lymphocyte count and all six CBC-derived IIBs discriminated pyogenic from *K. kingae* infection substantially better than most conventional inflammatory markers. Adding lymphocyte count or an individual IIB to the clinical model (age, admission temperature, and CRP) increased optimism-corrected AUCs from 0.848 to 0.951–0.962. The abnormal-IIB-count model achieved the highest corrected AUC of 0.970, although it did not significantly outperform the simpler lymphocyte-count model. These findings suggest that routine differential blood-cell information may provide useful complementary evidence when the causative organism is not yet known.

First, our results confirm that *K. kingae* OAIs showed a clearly distinct and milder inflammatory profile than pyogenic infections. In *K. kingae* OAIs, conventional infection markers were only mildly abnormal or remained within normal ranges, offering limited support for a typical OAI presentation. Among these conventional markers, CRP was the only one with good discriminatory performance (AUC 83%), whereas platelet count, ESR, and WBC count showed limited or no value for distinguishing between groups. These findings align with previous studies comparing OAIs caused by *K. kingae* with those due to MSSA or other pyogenic pathogens [4,5]. In an earlier study, we evaluated demographic, clinical, and biological parameters for their ability to distinguish *K. kingae* OAIs from MSSA infections. Among the evaluated criteria, only age showed excellent discriminatory performance between the two groups; CRP, platelet count, and temperature provided only fair to good accuracy [5]. Another study sought predictive criteria to distinguish *K. kingae* OAIs from infections caused by pyogenic pathogens in children younger than 4 years. Band shift and CRP showed excellent discriminatory performance, whereas WBC count showed good discriminatory value [4]. Our findings only partly support these results: CRP retained discriminatory value, whereas WBC count and band shift were less consistent.

A key finding of the present study is that blood cell analysis may have been underused. Although total WBC count was not informative, differential leukocyte counts—including neutrophils, lymphocytes, monocytes, and platelets—may provide valuable information for distinguishing OAI pathogens. Indeed, leukocyte subtype analysis revealed clear differences in discriminatory performance: lymphocyte count showed excellent discrimination between groups (AUC 93%, median 4-fold difference between *K. kingae* and pyogenic infections), surpassing even CRP, just as monocyte count showed good discrimination (AUC 88%). The limited discriminatory value of total WBC count likely reflects its nonspecificity and the marked age-related variation in paediatric leukocyte counts [30,31]. Because leukocyte counts are physiologically higher in young children, total WBC count alone may be inadequate for differentiating pathogens [26,27,28]. These results indicate that differential leukocyte components, particularly lymphocyte count, may be more informative than total WBC count and should be routinely examined in the initial evaluation of suspected OAI in young children.

The main contribution of this study is the first systematic evaluation of the potential value of systemic immune–inflammatory biomarkers for distinguishing OAIs caused by *K. kingae* from those caused by pyogenic bacteria in children younger than 5 years. In this cohort, all six IIBs (NLR, MLR, PLR, SII, SIRI, and PIV) showed excellent discriminatory performance (AUCs >90%) and outperformed most classical inflammatory markers. Their high sensitivities may be useful as complementary evidence when attempting not to miss potentially aggressive pyogenic infections. MLR and SIRI had a sensitivity of 100%, followed by NLR and PIV with 97%. As composite indices combining leukocyte subtypes and platelet parameters, these biomarkers may capture different aspects of the systemic inflammatory response. However, because they share overlapping cellular components and did not significantly outperform the simpler lymphocyte-count model, they should be regarded as complementary rather than independent or superior diagnostic signals. This finding is supported by recent evidence that NLR predicts the presence and severity of paediatric musculoskeletal infections more accurately than WBC count [31].

The substantial differences in median IIB values between *K. kingae* and pyogenic bacterial OAIs likely reflect fundamental differences in pathogen virulence and host inflammatory response. Pyogenic organisms, such as *S. aureus* and *streptococci*, typically induce a strong inflammatory reaction in OAIs, with laboratory findings consistent with severe invasive bacterial disease [30]. In contrast, *K. kingae* typically causes mild or subacute infections, with limited systemic inflammation and only modest increases in inflammatory markers [30].

As with conventional infection markers, the number of abnormal IIBs may provide clinically useful information. In this study, all six biomarkers exceeded the proposed thresholds in 90% of children with pyogenic bacterial OAIs, whereas 76% of children with *K. kingae* OAIs had no abnormal IIBs. However, this separation was not absolute. Twelve of 118 (10.2%) *K. kingae* infections had all six IIBs above the proposed thresholds, whereas 4 of 39 (10.3%) pyogenic infections did not have all six IIBs elevated. Importantly, none of the pyogenic infections had one or fewer abnormal IIBs. This overlap represents a clinically relevant diagnostic grey area and indicates that IIBs should not be used in isolation to determine bacterial aetiology. These indices should therefore be interpreted cautiously within a broader diagnostic framework that includes age, clinical presentation, fever, pain severity, infection site, imaging findings, microbiological results, and local epidemiology, as recommended by current PIDS/IDSA guidelines [30]. Early differentiation may nevertheless be clinically important because pyogenic OAIs generally produce a more aggressive inflammatory response and carry a greater risk of joint destruction, growth disturbance, and long-term functional impairment. Rapid recognition of a potentially pyogenic infection may therefore support timely invasive diagnostic procedures and appropriate antimicrobial treatment, particularly when an infection involves a developing joint or adjacent growth plate.

The multivariable analyses further showed that CBC-derived information provided substantial discriminatory value beyond age, admission temperature, and CRP. Adding an individual IIB increased the optimism-corrected AUC from 0.848 to between 0.951 and 0.962, and all extended models had significantly higher apparent AUCs than the clinical model (age, admission temperature, and CRP) after Holm adjustment. Lymphocyte count alone achieved a corrected AUC of 0.961, confirming that a substantial proportion of the discriminatory signal is already contained in the differential leukocyte count. The model incorporating the number of abnormal IIBs showed the highest observed performance, with a corrected AUC of 0.970; apparent sensitivity and specificity at the Youden-optimal threshold were 97.3% and 90.3%, respectively.

The cumulative number of abnormal IIBs may provide an intuitive summary of the concordant inflammatory pattern associated with pyogenic infections. Nevertheless, the abnormal-IIB-count model did not significantly outperform the simpler lymphocyte-count model. Moreover, because the six IIBs are calculated from overlapping leukocyte and platelet components, they should not be considered six independent diagnostic signals. Direct examination of the lymphocyte count may therefore offer a particularly simple initial indicator, while the number of abnormal IIBs may provide complementary evidence when several indices point in the same direction.

Previous diagnostic approaches have primarily combined age, admission temperature, CRP, WBC count, platelet count, or band count [4,5,32]. The present findings extend these approaches by demonstrating that differential leukocyte counts and CBC-derived IIBs provide information beyond age, fever, and CRP. However, these models remain exploratory and should not yet be considered validated bedside prediction tools.

This study has several strengths: a large cohort of *K. kingae* OAIs, a pyogenic bacterial OAI comparator group, systematic molecular testing with specific *K. kingae* PCR assays, and evaluation of biomarkers derived from routine laboratory tests, which may support clinical implementation. Additionally, the nearly 20-year study period for *K. kingae* cases (2007–2025) reflects contemporary diagnostic practices with molecular detection, enhancing generalisability to current clinical settings.

Several limitations should be acknowledged. The retrospective design may have led to missed cases and variability in clinical and laboratory assessment, particularly because blood samples were collected at different intervals after symptom onset. Some older records remained paper-based, which contributed to incomplete admission variables. Some infections, including spondylodiscitis, were excluded when bacteriological confirmation by aspiration was unavailable, despite strong clinical suspicion of *K. kingae* involvement. Sample sizes also differed for admission temperature and ESR because these variables were not available in all patients. In addition, the smaller pyogenic OAI cohort may limit the precision of estimates for this group. The different study periods for *K. kingae* (2007–2025) and pyogenic infections (1997–2025) may introduce temporal bias; however, restricting both groups to 2007–2025 produced materially similar IIB discrimination (AUCs 0.921–0.944 versus 0.919–0.940 in the primary analysis). Residual temporal bias nevertheless cannot be excluded. The pyogenic comparator also comprised a limited and uneven range of bacterial species, and inter-species or strain-level differences in virulence may generate different host inflammatory responses; consequently, the observed performance may not generalise to cohorts with a broader pyogenic pathogen spectrum. Because the power analysis was retrospective and based on observed effects, it should be interpreted descriptively and does not substitute for an a priori sample-size calculation. Finally, as the proposed biomarker cut-offs were derived from the same cohort, external validation is required before their integration into routine clinical algorithms. Furthermore, this single-centre study from a tertiary paediatric hospital may not reflect community practice patterns or regions with different *K. kingae* prevalence.

The multivariable models were evaluated in a complete-case population of 150 children, smaller than the population used for the individual CBC and IIB analyses because admission temperature was unavailable in 5 *K. kingae* and 2 pyogenic cases. Complete-case selection may therefore have introduced bias. Furthermore, the IIB thresholds and models were developed in the same single-centre cohort. Although bootstrap internal validation was performed and the complete threshold-selection process was repeated for the abnormal-IIB-count model, external validation remains necessary. The relatively small number of pyogenic infections also limits the precision of performance estimates and comparisons between the highest-performing models. Finally, because the six IIBs share several of the same cellular components, their combined count should not be interpreted as the accumulation of six independent predictors.

## 5. Conclusions

In conclusion, CBC-derived immune–inflammatory biomarkers showed substantial discriminatory information beyond conventional clinical markers in this cohort of young children with OAI. Individual IIBs showed excellent univariable performance, and adjusted models indicated that lymphocyte count and IIBs added information beyond age, admission temperature, and CRP. The model incorporating the number of abnormal IIBs showed the highest observed discrimination, although the model incorporating lymphocyte count alone achieved nearly equivalent performance and may represent a simpler clinical indicator. These findings remain exploratory, and the cumulative IIB count and probability models require external validation in larger and more bacteriologically diverse cohorts before they can be used to guide individual management decisions.

## Figures and Tables

**Table 1:** Epidemiology of study population for pyogenic and *K. kingae* OAIs.

	Pyogenic OAI (*n* = 39)	*K. kingae* OAI (*n* = 118)
Demographics		
Mean age months ± SD	24.7 ± 18.5	17.7 ± 8.4
Male sex	21 (53.8)	61 (51.7)
Female sex	18 (46.2)	57 (48.3)
Type of OAI, *n*		
Septic arthritis	13	69
Hip	6	7
Knee	3	44
Elbow	4	3
Shoulder	-	3
Ankle	-	4
Wrist	-	4
Other	-	4
Osteomyelitis (OM)	15	23
Long bones	11	13
Tarsal/carpal bones	1	8
Flat bones	2	-
Clavicle	1	2
Arthritis with concomitant OM	11	15
Spondylodiscitis	-	2
Other OAI	8	12

Note: OAI categories are not mutually exclusive; 47 OAI manifestations were recorded in the pyogenic group, and 121 in the *K. kingae* group. Percentages for OAI type used n = 47 and n = 121 as denominators. Percentages for anatomical sites are calculated within the corresponding OAI group.

**Table 2:** All markers for *K. kingae* and pyogenic OAIs with Wilcoxon test for children below the age of 5 years.

Marker	Median [IQR]		Wilcoxon Test	
	*K. kingae*	Pyogenic	*p*	ES
Admission T	37.0 [36.6–38.0]	38.3 [37.6–39.0]	<0.001	0.524
CRP	18.3 [6.7–32.0]	86.0 [41.0–125.5]	<0.001	0.649
ESR	35.0 [23.0–46.0]	51.0 [22.0–65.5]	0.096	0.214
WBC	12.3 [9.8–14.1]	13.3 [10.8–18.1]	0.085	0.185
Neutrophils	5.12 [3.75–6.89]	8.25 [5.69–11.0]	<0.001	0.482
Monocytes	0.891 [0.578–1.58]	3.45 [2.75–4.97]	<0.001	0.756
Lymphocytes	5.38 [3.45–6.48]	1.25 [0.582–1.58]	<0.001	−0.850
Thrombocytes	394.5 [332.3–491.3]	381.0 [290.0–512.5]	0.468	−0.078
NLR	1.02 [0.605–1.76]	6.82 [4.62–12.5]	<0.001	0.880
MLR	0.185 [0.107–0.291]	3.09 [1.82–7.71]	<0.001	0.845
PLR	79.7 [55.0–118.5]	326.6 [216.9–598.9]	<0.001	0.839
SII	413.6 [230.9–842.2]	2619.6 [1828.4–4888.8]	<0.001	0.860
SIRI	0.911 [0.419–2.01]	25.9 [15.3–45.2]	<0.001	0.860
PIV	323.8 [150.8–928.1]	8435.1 [5623.1–21,491.1]	<0.001	0.857

Note: Admission T, admission temperature (°C); CRP, C-reactive protein (mg/L); ESR, erythrocyte sedimentation rate mm/h; WBC, white blood cell count; NLR, neutrophil-to-lymphocyte ratio; MLR, monocyte-to-lymphocyte ratio; PLR, platelet-to-lymphocyte ratio; SII, systemic immune–inflammation index; SIRI, systemic inflammation response index; PIV, pan-immune–inflammation value. All cell counts are expressed in G/L (10^9^ cells/L). Admission temperature was available in 113 *K. kingae* and 37 pyogenic cases. CRP values were available in 118 *K. kingae* and 39 pyogenic cases, while ESR was available in 97 *K. kingae* and 26 pyogenic cases. ES denotes the rank-biserial correlation for the pyogenic group relative to the *K. kingae* group; positive values indicate higher values in the pyogenic group and negative values indicate lower values.

**Table 4:** Discriminatory performance of clinical and CBC-extended models for pyogenic osteoarticular infection.

Model	Apparent AUC, % [95% CI]	Optimism- Corrected AUC, %	Δ Corrected AUC vs. Clinical Model	Sp %	Sn %
Age + temperature + CRP	86.3 [78.1–94.6]	84.8	Reference	93.8	75.7
Clinical + NLR	96.1 [92.6–99.6]	95.2	+10.4	86.7	94.6
Clinical + MLR	95.9 [93.1–98.6]	95.1	+10.3	85.8	97.3
Clinical + PLR	96.4 [93.9–98.9]	95.4	+10.6	85.8	97.3
Clinical + SII	96.2 [93.0–99.4]	95.4	+10.6	85.8	94.6
Clinical + SIRI	96.8 [94.3–99.3]	96.0	+11.2	85.8	97.3
Clinical + PIV	96.8 [94.3–99.3]	96.2	+11.4	85.0	97.3
Clinical + lymphocytes	96.8 [94.5–99.1]	96.1	+11.3	89.4	94.6
Clinical + number of abnormal IIBs	97.7 [95.9–99.5]	97.0	+12.2	90.3	97.3

Note: Apparent AUC, development-sample area under the receiver operating characteristic curve with 95% CI; Optimism-corrected AUC, AUC after 1000 bootstrap repetitions; Δ corrected AUC vs. clinical model, percentage-point difference from the age + temperature + CRP reference model. Sp, specificity; Sn, sensitivity. Pyogenic infection was coded as the positive outcome. The clinical model was defined as age in months, admission temperature, and CRP; in model names, “Clinical” denotes this three-variable model. All models were evaluated in the same complete-case cohort of 150 children, comprising 37 pyogenic and 113 *K. kingae* infections. Individual IIBs were entered as continuous variables after transformation using ln(1 + value). The abnormal-IIB-count model included the number of IIBs exceeding their individual ROC-derived thresholds, ranging from 0 to 6. Sensitivity and specificity are apparent. Optimism-corrected AUCs were estimated using 1000 bootstrap repetitions. Changes in corrected AUC are expressed as percentage-point differences, not relative percentage changes.

## Data Availability

The data supporting the findings of this study are not openly available due to sensitivity concerns. They are accessible from the corresponding author upon reasonable request. The data are stored in a controlled-access repository at Geneva University Hospitals, Switzerland.

## Abbreviations

The following abbreviations are used in this manuscript:

OAI	Osteoarticular infections
IIB	Immune–inflammation biomarker
PCR	Polymerase chain reaction
CRP	C-reactive protein
ESR	Erythrocyte sedimentation rate
WBC	White blood cell count
NLR	Neutrophil-to-lymphocyte ratio
MLR	Monocyte-to-lymphocyte ratio
PLR	Platelet-to-lymphocyte ratio
SII	Systemic immune–inflammation index
SIRI	Systemic inflammation response index
PIV	Pan-immune–inflammation value

## Appendix A

**Table A1:** Paired DeLong comparisons of model AUCs.

Comparison	AUC, Model 1	AUC, Model 2	DeLong *p* Value	Holm- Adjusted *p* Value
Age + temperature + CRP − Clinical + lymphocytes	0.863	0.968	0.005	0.019
Age + temperature + CRP − Clinical + NLR	0.863	0.961	0.002	0.016
Age + temperature + CRP − Clinical + MLR	0.863	0.959	0.010	0.019
Age + temperature + CRP − Clinical + PLR	0.863	0.964	0.007	0.019
Age + temperature + CRP − Clinical + SII	0.863	0.962	0.002	0.016
Age + temperature + CRP − Clinical + SIRI	0.863	0.968	0.004	0.019
Age + temperature + CRP − Clinical + PIV	0.863	0.968	0.004	0.019
Age + temperature + CRP − Clinical + number of abnormal IIBs	0.863	0.977	0.002	0.016
Clinical + number of abnormal IIBs − Clinical + lymphocytes	0.977	0.968	0.166	0.831
Clinical + number of abnormal IIBs − Clinical + NLR	0.977	0.961	0.254	0.831
Clinical + number of abnormal IIBs − Clinical + MLR	0.977	0.959	0.044	0.308
Clinical + number of abnormal IIBs − Clinical + PLR	0.977	0.964	0.128	0.765
Clinical + number of abnormal IIBs − Clinical + SII	0.977	0.962	0.221	0.831
Clinical + number of abnormal IIBs − Clinical + SIRI	0.977	0.968	0.170	0.831
Clinical + number of abnormal IIBs − Clinical + PIV	0.977	0.968	0.191	0.831

Note: AUC, area under the receiver operating characteristic curve. DeLong *p* values compare paired AUCs. Holm adjustment was applied within each comparison family. In the model names, “Clinical” denotes the model incorporating age, admission temperature, and CRP.
